# Psychopathic callousness and perspective taking in pain processing: an ERP study

**DOI:** 10.1093/scan/nsae022

**Published:** 2024-03-05

**Authors:** Victoria Branchadell, Rosario Poy, Pablo Ribes-Guardiola, Pilar Segarra, Javier Moltó

**Affiliations:** Affective Neuroscience Lab, Department of Basic and Clinical Psychology, and Psychobiology, Universitat Jaume I, Castelló 12071, Spain; Affective Neuroscience Lab, Department of Basic and Clinical Psychology, and Psychobiology, Universitat Jaume I, Castelló 12071, Spain; Affective Neuroscience Lab, Department of Basic and Clinical Psychology, and Psychobiology, Universitat Jaume I, Castelló 12071, Spain; Affective Neuroscience Lab, Department of Basic and Clinical Psychology, and Psychobiology, Universitat Jaume I, Castelló 12071, Spain; Affective Neuroscience Lab, Department of Basic and Clinical Psychology, and Psychobiology, Universitat Jaume I, Castelló 12071, Spain

**Keywords:** psychopathy, callousness, pain processing, perspective taking, late positive potential

## Abstract

Psychopathy is a multifaceted personality disorder characterized by distinct affective/interpersonal traits, including callousness–unemotionality/meanness, which are often considered the hallmarks of empathic deficits. It has been posited that the processing of others’ pain could play an important role in empathy capabilities. This study aimed to investigate the influence of perspective taking on electrocortical responses during pain processing in relation to psychopathic callousness. The late positive potential (LPP) —a well-established electrophysiological indicator of sustained attention to motivationally significant stimuli— was measured while 100 female undergraduates viewed images depicting bodily injuries while adopting an imagine–self or an imagine–other perspective. Callousness factor scores —computed as regression-based component scores from EFA on three relevant self-report measures of this dimension— predicted reduced LPP amplitudes to pain pictures under the imagine–other (but not imagine–self) perspective, even after controlling for other LPP conditions. This result suggests that high-callous individuals exhibit diminished brain responsiveness to others’ distress, potentially contributing to the empathic deficits observed in psychopathy. This finding highlights the usefulness of the LPP and perspective taking in studies on pain processing to refine our understanding of the low empathy characteristics of psychopathy in biobehavioral terms.

## Introduction

Empathy is defined as the capability to understand and share the affective states of others, and plays a fundamental role in social interactions. It facilitates prosocial behaviors and inhibits antisocial or aggressive actions (Decety and Svetlova, 2012; Decety and Cowell, 2018). Impaired empathy can result in significant social disfunctions, which characterizes various forms of psychopathology. Psychopathy, a multifaceted personality disorder involving distinctive emotional, interpersonal and behavioral deviations, which is marked by symptom features such as callousness, lack of guilt and shallow affect (Cleckley, 1976; Hare and Neumann, 2008; Patrick and Bernat, 2009), can be regarded as the archetypal empathy disorder (Lockwood, 2016). Lack of empathy would explain the tendency of psychopathic individuals to harm and violate the rights of others and their lack of insight and remorse for their actions.

Consistent with the multifaceted perspective of psychopathy (Fowles and Dindo, 2009; Patrick and Bernat, 2009), impairments in empathic-emotional processing within psychopathy are particularly associated with its callousness–unemotionality/meanness traits (Campos *et al*., 2022, for a recent meta-analysis), which encompass phenotypic attributes such as lack of close attachments with others, emotional coldness and insensitivity, absence of guilt, and empowerment through cruelty (see, for example, Patrick and Bernat, 2009). Empirical studies have supported this relationship, demonstrating that psychopathy callousness traits —mainly assessed by the Meanness scale of the *Triarchic Psychopathy Measure* (TriPM; Patrick, 2010) and by the *Inventory of Callous-Unemotional traits* (ICU; Frick, 2004; Kimonis *et al*., 2008)— predict decreased recognition accuracy and blunted electrocortical responses to fearful faces (Brislin *et al*., 2018; Brislin and Patrick, 2019), reduced reactivity of the right amygdala to fear expressions (Viding *et al*., 2012), diminished potentiation of the noise-elicited startle reflex in response to violent films (Fanti *et al*., 2016), and reduced elaborative processing —as indexed by diminished amplitudes of the late positive potential (LPP) —of pictures depicting aggressive interactions (van Dongen *et al*., 2018) and task-relevant affective pictures (Ribes‐Guardiola *et al*., 2023).

Building on this, research on empathy deficits in psychopathy has also focused on pain empathy, hypothesizing that the brain’s pain network could play a crucial role in empathic capabilities (Decety, 2011). Others experiencing pain is a particularly significant signal, which can capture attention and promote caring and protective social functions. Therefore, responsiveness to others’ pain could serve as a valuable and ecologically valid indicator for empathic processing (Lamm *et al*., 2011). Paradigms involving pain experience have revealed higher pain tolerance in individuals exhibiting aggressive behavior (Niel *et al*., 2007) and psychopathic callousness traits (Miller *et al*., 2014; Brislin *et al*., 2016, 2022). These findings suggest that elevated pain thresholds may act as an underlying mechanism, which contributes to the underestimation of others’ pain experience and, consequently, insensitivity towards others’ distress. Regarding the concern for others’ pain, Caes *et al*. (2012), using a vicarious conditioning paradigm, found that psychopathic traits were associated with reduced defensive reactivity (as indexed by a reduced fear-potentiated startle responses) during the anticipation of painful stimulation in others, as well as a diminished ability to detect others’ pain. However, research focused on brain reactivity related to the distinct components of psychopathy has primarily used pain-viewing paradigms in which participants view images of hands and feet in painful or nonpainful situations while their brain activity is recorded. Neuroimaging studies have consistently reported a specific association between the selfish, callous and remorseless use of others component of psychopathy and reduced activation in key regions conforming the ‘pain matrix’ (such as the anterior insula, anterior cingulate cortex and/or amygdala; Decety, 2011) when participants adopt an other-perspective in which they imagine that the hand or foot in the picture belongs to someone else (Decety *et al*., 2013; Lockwood *et al*., 2013; Marsh *et al*., 2013; Seara-Cardoso *et al*., 2015). Interestingly, all these studies were conducted on samples composed exclusively of men —except Marsh *et al*. (2013), which used a mixed-gender sample but did not evaluate its influence— and the only study in a female sample found no significant relationships (Yoder *et al*., 2022). No callousness-related differences in the activation of these areas have been found under self-perspective conditions, when participants imagine that the hand or foot in the picture is their own (Decety *et al*., 2013; Marsh *et al*., 2013; Yoder *et al*., 2022). These results suggest that individuals with higher psychopathic callousness traits exhibit reduced brain activity in response to signals of distress in others, while maintaining typical activity levels when referring to themselves.

In addition to fMRI studies, research has also employed event-related potentials (ERPs) to characterize the temporal dynamics of pain empathy. A recent meta-analysis revealed that early and mid-latency components (< 300 ms) do not consistently show modulation in response to pain conditions, but reliable enhancements were observed in later components (P3/LPP) when comparing pain and no pain stimuli (Coll, 2018). The LPP is a sustained positive deflection in the ERP waveform, typically measured over centroparietal scalp regions, occurring between 400 and 1000 ms after stimulus presentation. It is a well-established ERP component associated with affective processing and has been theorized to reflect the sustained engagement of attention towards motivationally significant cues that activate the brain’s appetitive or aversive motivational systems (Hajcak and Foti, 2020). Considering this, the LPP appears to be a suitable electrocortical measure to investigate the effects of perspective taking on pain processing. However, limited research has explored this possibility thus far, with only one study reporting a greater differentiation between pain and no pain pictures in self-perspective conditions, but not in other-perspective conditions, when focusing on the early portion of this brain response (Li and Han, 2010).

Relevant to the current study, only two previous studies have examined pain processing in relation to psychopathic traits using ERPs. These studies have demonstrated callousness-related reductions in LPP amplitudes when participants viewed visual depictions of others in pain (Decety *et al*., 2015; Brislin *et al*., 2022). However, neither of these studies examined the potential moderating role of perspective taking. In one of these studies, Decety *et al*. (2015) presented participants with pictures of others’ hands and feet in painful situations and instructed them to either focus on the amount of concern they felt for the individuals, or the intensity of the pain the individuals in the pictures would experience. The authors found that psychopathic and callousness traits were associated with reduced LPP responses to pain stimuli only when participants were instructed to focus on their level of concern for others. This provides evidence for specific impairments in the capacity for empathic concern when processing distress signals in others at the electrophysiological level. Furthermore, psychopathic and callousness traits were associated with lower ratings of both empathic concern and pain intensity in this study.

In the second study, Brislin *et al*. (2022) found that meanness/callousness traits of psychopathy predicted reduced LPP amplitudes in response to pictures of others in pain during a passive viewing task without specific perspective-taking instructions. Additionally, these traits were associated with lower ratings of pain intensity in both self- and other-perspective conditions. Unfortunately, this study did not investigate whether the blunted electrocortical processing of pain in individuals with higher callousness may be differentially modulated by the adopted perspective.

## The current study

To obtain a more comprehensive understanding of callousness-related differences in pain processing at the electrophysiological level, this study aimed to investigate, for the first time, the influence of perspective taking on LPP amplitudes elicited by pain pictures in relation to psychopathic callousness traits. To achieve this objective, EEG data were recorded while a sample of female undergraduates viewed pictures depicting bodily injuries while imagining that the person in the picture was either themself (self-perspective) or an unknown other (other-perspective). Callousness traits were assessed using a multimeasurement approach, by extracting scores on a factor index of this trait dimension using three self-report scales that have been demonstrated to be suitable indicators of the callousness traits of psychopathy (see Drislane *et al*., 2014), and that have also been used in prior research in pain empathy (Lockwood *et al*., 2013; Decety *et al*., 2015; Brislin *et al*., 2022): the TriPM Meanness scale, the Primary Psychopathy scale of the *Levenson Self-Report Psychopathy Scale* (LSRP; Levenson *et al*., 1995) and the ICU.

Building upon prior evidence demonstrating associations between the callousness traits of psychopathy and reduced brain reactivity —both with fMRI and EEG measures— to pain in others (e.g. Decety *et al*., 2013, 2015; Marsh *et al*., 2013), it was hypothesized that Callousness factor scores would be specifically correlated with reduced LPP amplitudes to pain stimuli under the other-perspective but not under the self-perspective viewing instructions.

## Method

### Participants

The initial sample consisted of 105 female undergraduates recruited from the Universitat Jaume I of Castellón (Spain). Before the experimental session, five participants were excluded from the study because they were undergoing psychiatric or pharmacological treatment at the time of the experiment. The final sample comprised a total of 100 participants, ranging in age from 18 to 35 years (*M* = 19.44, *SD* = 2.6). The experimental research procedures were approved by the Ethical Committee of the Universitat Jaume I and adhered to the ethical principles for human research outlined in the Declaration of Helsinki. Written informed consent was obtained from all the participants and they received academic credit as compensation for their participation.

### Self-report measures

The *Triarchic Psychopathy Measure* (TriPM; Patrick, 2010; Spanish version, Poy *et al*., 2014) is a questionnaire specifically developed to assess the three trait dimensions proposed in the triarchic model of psychopathy (Patrick and Bernat, 2009). The TriPM Meanness scale measures lack of empathy and remorse, scorn for and absence of close attachments with other people, defiance of rules and cruelty (e.g. ‘I’ve injured people to see them in pain’). It consists of 19 items that are answered using a 4-point Likert scale (0 = false, 1 = somewhat false, 2 = somewhat true, 3 = true).

The *Levenson Self-Report Psychopathy Scale* (LSRP; Levenson *et al*., 1995; Spanish version, Andreu-Rodríguez *et al*., 2018) was developed to assess both factors of *Hare’s Psychopathy Checklist-Revised* (PCL-R; Hare, 2003) in non-institutionalized young adult samples. The 16-item LSRP Primary scale measures tendencies towards deception and manipulation, lack of guilt and emotional coldness or insensitivity (e.g. ‘‘Success is based on survival of the fittest; I am not concerned about the losers”). Each item is ranked on a 4-point Likert scale (1 = disagree strongly, 2 = disagree somewhat, 3 = agree somewhat and 4 = agree strongly).

The *Inventory of Callous-Unemotional Traits* (ICU; Frick, 2004; Kimonis *et al*., 2008; Spanish version, Ezpeleta *et al*., 2013) is a 24-item questionnaire specifically developed to assess the construct of callous unemotionality in individuals across various age groups, including children, adolescents and young adults (see also Byrd *et al*., 2013; Kimonis *et al*., 2013; Drislane *et al*., 2014), encompassing traits such as carelessness and lack of emotional responsiveness (e.g. ‘I do not feel remorseful when I do something wrong’). Items are rated on a 4-point Likert scale, from 0 (not at all true) to 3 (definitely true).

All three scales demonstrated good internal consistency reliability in the current sample, with Cronbach’s α coefficients being 0.72 for TriPM Meanness, 0.78 for LSRP Primary and 0.83 for ICU. Descriptive statistics (mean, standard deviation, range) for the scores of these scales are presented in Table 1. An exploratory factor analysis (EFA) on scores from these scales was conducted to obtain a general Callousness factor representing the shared variance between the different measures. Results of the principal-axis factor analysis (Barlett’s *χ*^2^ = 88.5, *P* < 0.001; Kaiser-Meyer-Olkin = 0.693) and the parallel analysis revealed one single factor (eigenvalue = 1.69) accounting for 56.3% of the total variance. The factor loadings were 0.82, 0.77 and 0.66 for the TriPM Meanness, ICU and LSRP Primary scales, respectively. The regression-based estimation method was used to compute a factor score for each participant, reflecting the sum of beta-weighted scores on the three callousness measures.

**Table 1.** Descriptive statistics for self-report and ERP data for the overall sample (*N* = 100)

Variable	M (SD)	Min.	Max.
***Self-report data***			
TriPM Meanness	9.28 (5.44)	1	28
LSRP Primary	27.52(6.03)	18	40
ICU	18.42 (8.17)	4	40
***ERP data***			
LPP Pain Self	1.23 (1.10)	−0.86	4.92
LPP Pain Other	1.21 (1.04)	−1.22	4.49
LPP No Pain Self	1.12 (0.96)	−0.63	3.87
LPP No Pain Other	0.86 (0.89)	−0.81	4.52

*Note*. TriPM = Triarchic Psychopathy Measure (Patrick, 2010); LSRP = Levenson Self-Report Psychopathy Scale (Levenson *et al*., 1995); ICU = Inventory of Callous Unemotional Traits (Frick, 2004).

### Procedure and experimental task

Before the experimental session, participants completed the self-report measures in group sessions; only the anonymized data were stored. The experimental session was conducted individually in an isolated and dimly lit room. Participants were seated 110 cm away from a monitor screen where stimuli (horizontal and vertical visual angle of 7.28° and 5.21°) were displayed. Presentation® v.20.1 software (Neurobehavioral Systems, Inc. Albany, CA, USA) was used to control the order, sequence and timing of stimulus presentations on a PC Pentium Core 2 Duo (Intel) computer. During EEG recording, participants viewed a total of 128 pictures depicting hands and feet of individuals in both painful and non-painful everyday situations (Jackson *et al*., 2005) —for example, cutting a cucumber with a finger under the knife (pain) or without the finger under the knife (no pain). Each picture was presented twice with two different perspectives. Participants were instructed to adopt either a self-perspective (‘imagine the person in the picture is you’) or other-perspective (‘imagine the person in the picture is someone unknown’), while viewing the pictures within each block of stimuli.

Each trial began with a fixation cross displayed on the screen for a duration of 2000, 2500 or 3000 ms, followed by the presentation of a pain or no pain (neutral) picture for 1500 ms. The task consisted of eight blocks, each containing 32 trials, resulting in a total of 256 trials. The pictures were randomly presented within each block and the perspective instruction changed between consecutive blocks, with a 30 s rest period between blocks. The overall duration of the task, including eight practice trials and breaks, was ∼22 min.

Two subsamples of participants (*n* = 18 for half of the pictures; *n* = 13 for the other half) rated the arousal and the intensity of pain depicted in each picture on scales ranging from 1 to 9, under both the self-perspective and other-perspective instructions. The results confirmed higher ratings for *pain* (*M*_arousal_ = 6.18; *M*_pain_ = 6.22) compared to *no pain* pictures (*M*_arousal_ = 2.50; *M*_pain_ = 1.18; *F’*s > 298.77; *P*’s < 0.001), and for *self*- (*M*_arousal_ = 4.66; *M*_pain_ = 4.46) compared to *other-perspective* instructions (*M*_arousal_ = 4.01; *M*_pain_ = 3.57; *F*’s > 39.12; *P*’s < 0.001). No significant interactions were found (*F*’s < 3.31; *P*’s > 0.079).

### Psychophysiological recording and data reduction

EEG activity was recorded from 257 electrodes using an Electrical Geodesic (EGI; OR, USA) high-density EEG system. The signals were amplified and filtered (analog filters: 0.10–100 Hz bandpass) with a NetAmps 400 amplifier system with NetStation v5.4.1.2 installed on a MacBook Pro (Apple) computer. The EEG data were continuously digitized at a sampling rate of 250 Hz using a 24-bit analog-to-digital converter. The reference electrode was placed on the vertex scalp site (Cz) and the scalp impedances were kept < 50 kΩ, following the manufacturer’s guidelines.

Offline preprocessing of the raw EEG data was performed using Brain Electrical Source Analysis software (BESA v7.1.2.1; MEGIS software GmbH, Germany). Visual inspection of the raw recordings was performed to identify and interpolate data for bad electrodes. Eyeblink (EOG) and electrocardiogram (EKG) artifacts in the continuous EEG data were manually corrected using a principal component analysis-based adaptive artifact-correction method in Brain Electrical Source Analysis. The artifact-corrected data were then subjected to a low-pass filter with a cutoff frequency of 30 Hz. Stimulus-synchronized epochs were extracted from −200 to +1000 ms after the picture onset and a baseline correction was applied using the 200 ms period preceding the stimulus onset. A semi-automated procedure was then used to detect and reject epochs containing amplitude deflections exceeding 75 μV between successive sampling points or surpassing an amplitude threshold of 120 μV. Additionally, epochs with a low signal threshold of 0.01 μV were discarded. The accepted epochs were subsequently converted to the average reference.

### ERP measurement

For each participant, separate ERP averages were computed for each sensor and condition. The LPP was scored as the mean amplitude of a 14-sensor centroparietal cluster (EGI sensors: 45, 79, 80, 81, 89, 90, 100, 101, 129, 130, 131, 132, 143 and 257; see Ribes‐Guardiola *et al*., 2023, for the same electrode configuration) during a 400–1000 ms time window following stimulus onset. This time window was selected based on prior research investigating LPP amplitudes for pain pictures in relation to callousness (Decety *et al*., 2015; Brislin *et al*., 2022).

The means (and standard deviations) of total valid trials per condition were 49.28 (12.98) for Pain Self, 49.73 (11.20) for Pain Other, 51.63 (9.27) for No Pain Self and 48.81 (12.05) for No Pain Other. The reliability of LPP amplitudes, assessed using split-half (odd-even method) correlations adjusted for attenuation using the Spearman–Brown prophecy formula, was found to be moderate-to-high for all conditions: 0.77 for LPP Pain Self, 0.68 for LPP Pain Other, 0.78 for LPP No Pain Self and 0.70 for LPP No Pain Other.

**Fig. 1.**
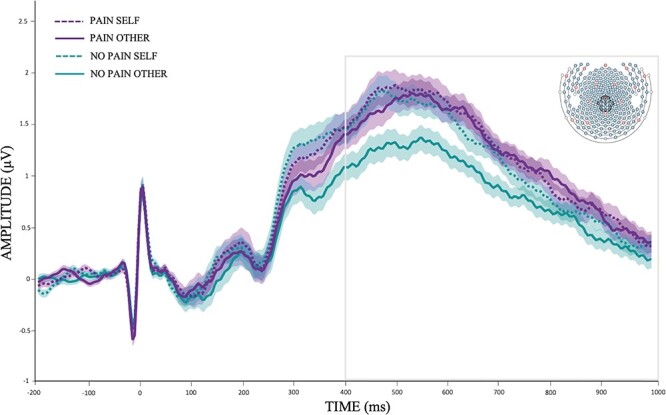
Grand average event-related potentials waveforms for pain (purple) and no pain (green) under self-perspective (dotted lines) and other-perspective instructions (solid lines) at the centroparietal sensor cluster (EGI sensors: 45, 79, 80, 81, 89, 90, 100, 101, 129, 130, 131, 132, 143 and 257).

Upon visual inspection of the grand averaged waveforms, a trigger-related artifact was observed around the EEG ground electrode affecting electrodes at central parietal locations (see Figure 1). This artifact coincided with the timing of the trigger codes for visual stimuli sent by the Presentation software to the acquisition software. The magnitude of the artifact did not vary across conditions, and thus did not affect the results or their interpretation. To further investigate whether the presence of this artifact-related activity affected the results reported using time-windowed analyses, we conducted a temporal Principal Component Analysis (PCA; Dien, 2012) on the averaged epochs (−200 to 1000 ms) at the centroparietal cluster. This allowed us to separate the LPP component from the influence of other components. The results of these analyses are presented in the Supplemental Material, showing the same pattern of results reported in the main text.

### Statistical analyses

The data analyses were conducted using Jamovi 2.3.21.0 software (The Jamovi Project, Sydney, Australia). First, to validate the selected task procedure, the effects of pain and perspective on LPP amplitudes were tested by conducting a 2 × 2 repeated measures ANOVA with *Pain* (pain, no pain) and *Perspective* (self, other) as within-subjects factors. Significant interaction effects were further explored using post-hoc comparisons.

Second, to investigate the relationships between LPP responses and callousness, bivariate Pearson’s *r* correlations were calculated between LPP amplitudes and callousness scores (omnibus component and individual scale scores). In addition, in order to clarify predicted specific effects of Callousness factor scores on LPP reactivity to pain in the other-perspective condition, residual LPP Pain Other amplitudes were computed. This residual method allows to isolate neural activity for LPP Pain Other specific to this condition after accounting for its overlap with LPP amplitudes elicited in task conditions involving non-painful scenarios or the adoption of a self-oriented perspective (see Meyer *et al*., 2017). Thus, unstandardized residuals were saved from a regression model on which LPP Pain Other amplitudes served as the criterion, and the three remaining LPP conditions acted as concurrent predictors. This resulting variable was then correlated with Callousness factor scores.

## Results

### Task effects

Descriptive statistics for LPP amplitudes are shown in Table 1. The ANOVA revealed significant main effects of *Pain, F*(1, 99) = 11.72, *P* < 0.001; *η*_p_^2^ = 0.11, and *Perspective, F*(1, 99) = 5.90, *P* = 0.017, *η*_p_^2^ = 0.06, indicating that LPP amplitudes were larger for pain than for no pain pictures (1.22 *vs* 0.99 μV, respectively), and in the self- than in the other-perspective condition (1.18 *vs* 1.03 μV, respectively). Furthermore, there was a significant *Pain *× *Perspective* interaction, *F* (1, 99) = 3.98, *P *= 0.049, *η*_p_^2^ = 0.04. Post-hoc comparisons revealed larger LPP amplitudes for pain than for no pain pictures only in the other-perspective condition, *t*(99) = 3.56, *P* < 0.001 (*P* = 0.202 for the self-perspective condition; see Figure 1).

### Callousness effects

**Fig. 2.**
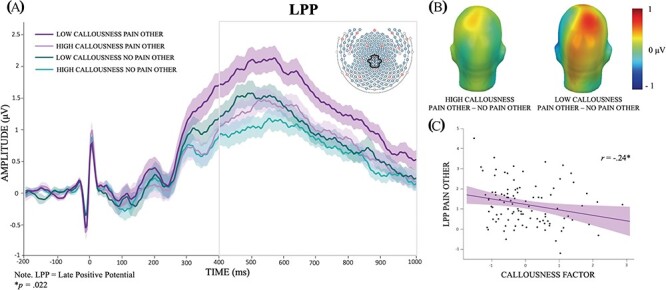
Relationship between LPP amplitudes for other-perspective pain and no pain pictures and Callousness factor scores. (A) Grand average event-related potentials waveforms for the other-perspective pain (purple) and no pain (green) conditions at the centro-parietal cluster for participants on the bottom (dark) and top (light) median-split groups on the Callousness factor. (B) LPP scalp distribution differences between pain and no pain conditions for the 400–1000 ms time window. (C) Scatterplot depicting the correlation between Callousness factor scores and LPP amplitudes for other-perspective pain pictures.

Bivariate Pearson’s correlations between callousness measures and LPP amplitudes can be found in Table 2. Callousness factor scores (as well as ICU scores) showed a significant negative correlation with LPP amplitudes for pain pictures in the other-perspective condition (*r*’s > −0.24, *P*’s < 0.02). In addition, when considering LPP Pain Other residual scores, the correlational analysis still showed a negative association with Callousness factor scores, *r *= −0.21, *P* = 0.036, thus corroborating a specific association between callousness and the unique variance in LPP Pain Other amplitudes. Figure 2 visually illustrates this result, showing the grand averaged waveforms for median-split groups on Callousness factor scores (Figure 2A), the LPP scalp distribution (Figure 2B) and the scatterplot depicting the association between Callousness factor scores and LPP amplitudes for pain and no pain pictures under the other-perspective viewing instruction (Figure 2C).

**Table 2.** Bivariate Pearson correlations between self-report and ERP data for the overall sample (*N* = 100)

**Variable**	**1**	**2**	**3**	**4**	**5**	**6**	**7**	**8**
***Self-Report data***								
1. ICU	*–*							
2. LSRP Primary	0.50***	–						
3. TriPM Meanness	0.63***	0.54***	–					
4. Callousness factor	0.85***	0.73***	0.91***	–				
***ERP data***								
5. LPP Pain Self	−0.21*	−0.04	−0.11	−0.15	–			
6. LPP Pain Other	−0.25*	−0.14	−0.19	−0.24*	0.70***	–		
7. LPP No Pain Self	−0.13	0.06	−0.06	−0.07	0.70***	0.66***	–	
8. LPP No Pain Other	−0.18	−0.08	−0.08	−0.13	0.59***	0.50***	0.58***	–

*Note*. TriPM = Triarchic Psychopathy Measure (Patrick, 2010); LSRP = Levenson Self-Report Psychopathy Scale (Levenson et al., 2995); ICU = Inventory of Callous-Unemotional Traits (Frick, 2004).

* *P < *0.05, ** *P < *0.01, *** *P < *0.001.

## Discussion

The present study aimed to examine, for the first time, the influence of perspective taking on electrocortical processing of pain in relation to the callousness traits of psychopathy. Consistent with our hypothesis, individuals with higher levels of callousness exhibited reduced LPP amplitudes for pain pictures when imagining someone else (and not themself) in the painful situation. This finding suggests that callousness traits of psychopathy are related to a blunted elaborative processing of distress cues in others, indicative of impaired empathic responding.

Prior research on fMRI has widely investigated the differences in brain reactivity between pain and no pain stimuli (Jackson *et al*., 2005; Gu and Han, 2007), revealing a more extensive activation of areas conforming the pain network during self- compared to other-perspective conditions (Jackson *et al*., 2006a,b; Lamm *et al*., 2007). Meta-analysis evidence on pain empathy ERPs has also demonstrated higher brain reactivity to pain compared to neutral stimuli in late components (Coll, 2018), fitting with our results which show higher LPP amplitudes for pain-related pictures. However, there is limited research examining how ERP components related to pain processing are modulated by perspective taking. Thus, our study makes a significant contribution to electrocortical research on pain processing by incorporating perspective taking, enabling the differentiation of the empathic distress response elicited by others’ pain from the perception of one’s own pain experience. Furthermore, regardless of the picture type (i.e. pain or no pain), self-perspective consistently elicited greater LPP amplitudes than other-perspective instructions, indicating an enhanced sustained allocation of attention towards self-referenced stimuli. Interestingly, the LPP amplitude differentiation between pain and no pain pictures was observed only when participants imagined someone else in the painful situation, but not when they imagined themselves in the same situation. This finding contrasts with the only other study to date that has examined the perspective-taking modulation of electrocortical responses to perceived pain (Li and Han, 2010). Our results can be interpreted from the theoretical perspective of the LPP as an index of stimulus significance (Bradley, 2009; Hajcak and Foti, 2020). In our sample, the self-relevant condition enhanced brain reactivity regardless of the stimulus content, while the condition involving someone else allowed to differentiate pain *vs* no pain responses, with another person in a non-painful situation being the less motivationally relevant condition in electrocortical terms. Although more EEG studies examining the influence of perspective taking are needed, our results seem to suggest that the LPP can be considered as a valuable electrocortical measure to study neural deficits in pain empathy capacity as a function of perspective.

Indeed, by explicitly instructing participants to adopt either a self- or other-oriented perspective towards pain pictures, it was possible to confirm the blunted neural responsiveness to others’ distress that is theoretically linked to the callousness traits of psychopathy. This result represents the main contribution of the current study. As hypothesized, we found that callousness scores were associated with reduced LPP amplitudes to pain pictures when adopting an imagine-other perspective, even after controlling for the remaining conditions. These findings are consistent with prior evidence on callousness traits which has demonstrated reduced amplitudes of the LPP in conditions involving empathic concern (Decety *et al*., 2015) and reduced activation of brain regions associated with empathy for pain (Decety *et al*., 2013; Lockwood *et al*., 2013; Marsh *et al*., 2013; Seara-Cardoso *et al*., 2015). Taken together, these results suggest that diminished neural responses to others’ distress may be linked to the lack of concern and disdain for others that characterize psychopathic meanness/callousness. Given that neural reactivity at this fundamental level can facilitate affiliative behaviors (Decety and Svetlova, 2012), the absence of such reactivity may contribute to the callousness–unemotionality traits of psychopathy.

Our results must also be considered in light of prior work reporting significant associations between callousness traits and lower ratings of pain intensity under self- and other-oriented perspectives (Brislin *et al*., 2022). In contrast, our results show that the LPP seems to be more sensitive to the influence of perspective taking on pain processing in relation to callousness traits. Considered together, it seems that, for high-callous individuals, pain stimuli are perceived as less intense in general, regardless of their own involvement (i.e. self or other; see Brislin *et al*., 2022) —being this latter result consistent with evidence indicative of the higher pain tolerance of higher callous individuals (Miller *et al*., 2014; Brislin *et al*., 2016, 2022)— but its motivational relevance is diminished only when affecting someone else. The lack of convergence of measures from different modalities (self-report, electrophysiological) could be explained by the aspect of pain processing that each of them captures: subjective quantification of perceived pain (ratings) *vs* stimulus significance (LPP modulation). Brislin *et al*. (2022) did not find significant correlations between pain intensity ratings under self- and other-perspectives and LPP amplitudes to pain stimuli during passive picture viewing, suggesting that these two types of measures might assess different aspects of pain processing. Unfortunately, we did not collect ratings of pain intensity and arousal in the overall sample, but only in a small subsample in order to characterize relevant subjective dimensions (pain intensity, arousal) on which the stimuli employed to elicit electrocortical responses were expected to vary. In this regard, it would be highly informative for future studies on electrocortical processing of pain to obtain ratings assessing the stimulus significance/relevance from self- and other-perspectives, in addition to arousal and intensity, to complement evidence about electrocortical responsiveness to others’ distress.

Another notable contribution of our work is that the reduced reactivity of the LPP to others’ pain was found to be related to callousness traits, as indexed through a multimeasurement approach, which offers some advantages. Alternative measures developed to operationalize callousness–unemotionality/meanness show moderate correlations and, though converging in the assessment of the core traits subsumed in this construct, they also diverge by assessing other less central traits (Viding and Kimonis, 2018). For example, some measures assess lack of interest in one’s own performance (e.g. ICU: ‘I do not care about doing things well’), concern for material gains (e.g. LSRP Primary scale: ‘My main purpose in life is getting as many goodies as I can’) or sensation seeking (e.g. TriPM Meanness: ‘Things are more fun if a little danger is involved’). In fact, the scale scores used in our EFA did not equally contribute to the general Callousness factor. By using a single omnibus factor that encompassed the common variance across alternative operationalizations of the callousness–unemotionality/meanness dimension, we obtained a theoretically valid measure that de-emphasized unique and error variance associated with each instrument. This approach allowed us to confirm that it is the shared variance among these alternative scales, rather than the unique variance of each of them, that relates to reduced neural responsiveness to others’ pain (see Supplemental Material for exploratory analyses showing that none of the individual scale scores of callousness significantly related to LPP reactivity to others’ pain when controlling for the general Callousness factor).

In future work, it would be valuable to extend this approach to examine how alternative self-report indicators of callousness relate to other established physiological and behavioral indicators of affective processing of distress cues in others, such as reduced amygdala reactivity to fearful faces (Viding *et al*., 2012), diminished early ERP amplitudes and recognition accuracy to fear expressions (Brislin *et al*., 2018; Brislin and Patrick, 2019) or reduced elaborative processing of aggressive interactions (van Dongen *et al*., 2018). Systematically, studying patterns of covariance among these established physiological and behavioral indicators would be needed to refine our understanding of the biobehavioral processes linked to callousness traits (cf. affiliative capacity; Patrick, 2022; Patrick *et al*., 2019; see also Palumbo *et al*., 2020).

Some limitations of the current study should be acknowledged. First, our sample consisted exclusively of women, which may limit the generalizability of our findings. Although previous research has not found gender effects on reductions in LPP for pain pictures related to meanness/callousness (Decety *et al*., 2015; Brislin *et al*., 2022), follow-up studies using mixed-gender samples are needed to confirm that the association between electrocortical responsiveness to others’ pain and callousness is not gender-dependent. Furthermore, our undergraduate unselected sample showed a restricted range of callousness scores, which might have potentially attenuated the effects found. Therefore, in future studies, it would be valuable to preselect the sample based on callousness scores to ensure a better representation of high scores and allow for more robust effects to be observed. Additionally, considering the lack of convergence between self-report and electrophysiological measures of pain reactivity (as discussed earlier), it would be beneficial to complement the EEG measurements with subjective ratings of pain intensity, arousal and stimuli relevance. Finally, it would be interesting to incorporate EEG measurements (e.g. LPP) in more ecological and realistic tasks to study pain reactivity, such as vicarious pain paradigms that have demonstrated psychopathy-related differences in defensive reactivity and pain perception (e.g. Caes *et al*., 2012).

Despite these limitations, our study provides further evidence regarding the association between callousness–unemotionality/meanness traits and the processing of pain in others. These findings contribute to better understanding of empathy deficits, which have been long linked to the selfish and remorseless use of others, a symptom of psychopathy. Finally, our results highlight the utility of perspective-taking in electrocortical research on pain responsiveness, providing support for the use of the LPP as an indicator that has the potential to elucidate the biobehavioral processes associated with the meanness/callousness traits of psychopathy.

## Supplementary Material

nsae022_Supp

## Data Availability

The data underlying this article will be shared on reasonable request to the corresponding author.
