# Supplementary material for: Psychopathic callousness and perspective taking in pain processing: an ERP study
Source: Soc Cogn Affect Neurosci. 2024 Mar 5;19(1):nsae022. doi: 10.1093/scan/nsae022 (PMC10972532; doi:10.1093/scan/nsae022)
Supplement: nsae022_Supp [file nsae022_supp.zip › scan-23-155-File006.docx]

**Psychopathic Callousness and Perspective Taking in Pain Processing: an ERP study**

***Supplemental Material***

*Contents*

**1. LPP analyses using Principal Component Analysis (PCA)**

*p. 3. Supplemental Table 1. Bivariate Pearson Correlations between Self-report and ERP Data for the Overall Sample (N = 100)*

*p. 2. Supplemental Figure 1.* *Grand Average Event-Related Potentials Waveforms for Pain (Purple) and No Pain (Green) under Self-Perspective (Dotted Lines) and Other-Perspective (Solid Lines)*

*p. 4. Supplemental Figure 2. Grand Average Event-Related Potentials Waveforms for the Other-Perspective Pain Condition for Participants on the Bottom (Dark Purple) and Top (Light Purple) Median-Split Groups on the Callousness Factor*

**2. Partial correlations between individual scale scores of callousness and LPP Pain Other controlling for Callousness factor scores**

**Supplemental References**

p. 5

**1. LPP Analyses using Principal Component Analysis (PCA)**

To isolate the Late Positive Potential (LPP) amplitude and remove the artifact, the LPP was calculated using temporal Principal Component Analysis (PCA; Dien, 2012; Dien & Frinschkoff, 2005) on the channels of interest (i.e., the 14-sensor centroparietal cluster; see ERP measurement on the Method section). PCA is a statistical technique that identifies linear combinations of data points that capture consistent patterns of electrocortical activity (Foti et al., 2009).

Given that the artifact occurred when stimulus was presented (i.e., at time point 0), while the LPP is a later potential, PCA can effectively separate these two types of EEG signals. We used the ERP PCA Toolkit version 2.93 (Dien, 2010) to perform a temporal PCA with Promax rotation on the 300 timepoints in the data, considering all subjects and conditions. The covariance matrix and Kaiser normalization were used, as described in Dien et al. (2005). After examining the resulting Scree plot, nine temporal factors were extracted for rotation. The LPP becomes evident on the second factor at 484 ms (see Supplemental Figure 1).

**Supplemental Figure 1.** *Grand Average Event-Related Potentials Waveforms for Pain (Purple) and No Pain (Green) under Self-Perspective (Dotted Lines) and Other-Perspective (Solid Lines)*

**
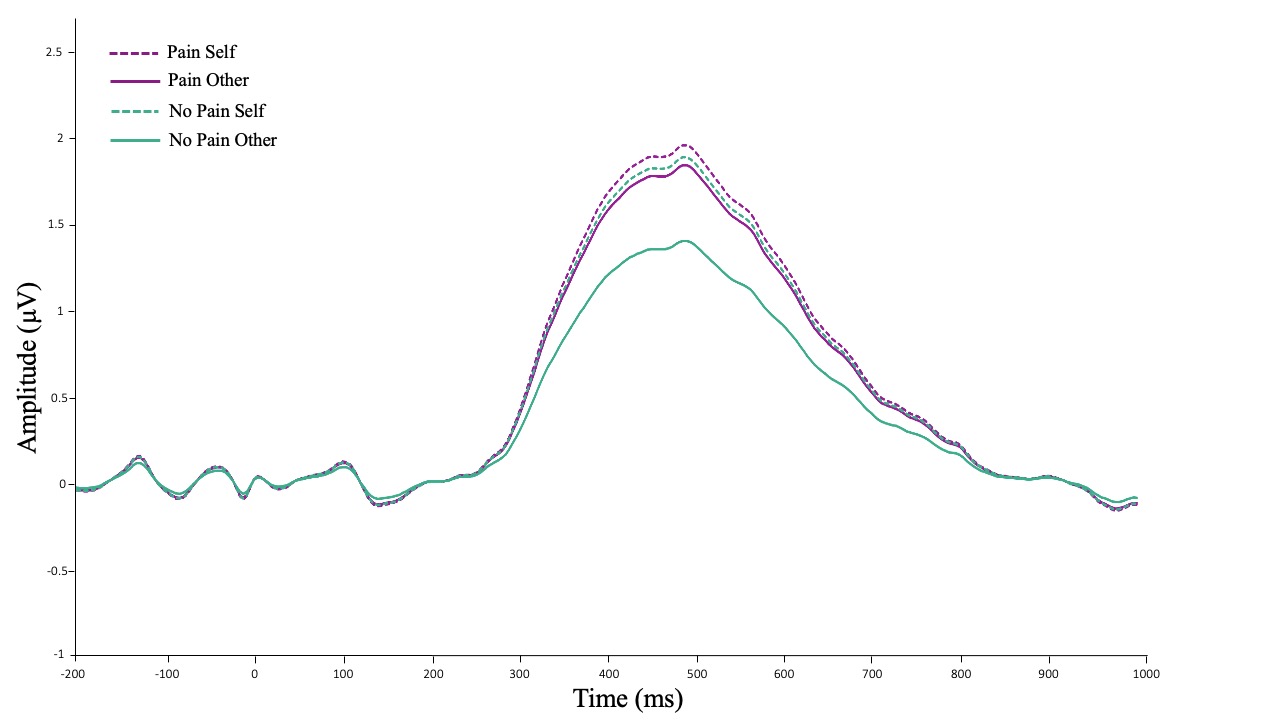
**

The results obtained from the LPP calculated using PCA are presented below:

Regarding **task effects**, a general linear model (GLM) analysis revealed significant main effects of *Pain*, *F*(1, 99) = 12.06, *p* < .001; *η*_p_^2^ = .11, ε = 1, indicating larger overall amplitudes for pain pictures compared to no pain pictures, and *Perspective*, *F*(1, 99) = 19.62, *p* < .001, *η*_p_^2^ = .17, ε = 1, indicating larger LPP overall amplitudes under the self-perspective compared to the other-perspective instruction. A significant *Pain* × *Perspective* interaction was also observed, *F* (1, 99) = 7.42, *p* = .008, *η*_p_^2^ = .07, ε = 1, revealing that LPP amplitudes were larger for pain pictures than for no pain pictures only under the other-perspective instruction, *t*(99) = 4.26, *p* < .001 (see Supplemental Figure 1).

Regarding the **effects** of **callousness**, a significant negative relationship was found between Callousness factor scores and LPP amplitudes for pain pictures in the other-perspective condition (*rs* > -.23, *ps* < .02; see Supplemental Table 1).

**Supplemental Table 1.** *Bivariate Pearson Correlations between Self-report and ERP Data for the Overall Sample (N = 100)*

| **Variable** | *1* | *2* | *3* | *4* | *5* | *6* | *7* | *8* |
| --- | --- | --- | --- | --- | --- | --- | --- | --- |
| ***Self-Report Data*** |  |  |  |  |  |  |  |  |
| *1. ICU* | - |  |  |  |  |  |  |  |
| *2. LSRP Primary* | .50*** | - |  |  |  |  |  |  |
| *3. TriPM Meanness* | .63*** | .54*** | - |  |  |  |  |  |
| *4. Callousness Factor* | .85*** | .73*** | .91*** | - |  |  |  |  |
| ***ERP Data*** |  |  |  |  |  |  |  |  |
| *5. LPP Pain Self* | -.17 | -.04 | -.11 | -.14 | - |  |  |  |
| *6. LPP Pain Other* | -.25* | -.12 | -.19 | -.23* | .85*** | - |  |  |
| *7. LPP No Pain Self* | -.14 | .08 | -.07 | -.07 | .81*** | .79*** | - |  |
| *8. LPP No Pain Other* | -.19 | -.08 | -.11 | -.16 | .76*** | .73*** | .72*** | - |

Note. TriPM = Triarchic Psychopathy Measure (Patrick, 2010); LSRP = Levenson Self-Report Psychopathy Scale (Levenson et al., 2995); ICU = Inventory of Callous-Unemotional Traits (Frick, 2004); LPP = Late Positive Potential

**p <* .05, ** *p <* .01, *** *p <* .001

In addition, this association remained significant when considering LPP Pain Other residual scores (computed by saving the unstandardized residuals from a regression model on which LPP Pain Other amplitudes served as the criterion, and the three remaining LPP conditions acted as concurrent predictors), *r* = -.23, *p* = .022, thus corroborating a specific association between callousness and the unique variance in LPP Pain Other amplitudes. Supplemental Figure 2 illustrates this finding.

**Supplemental Figure 2.** *Grand Average Event-Related Potentials Waveforms for the Other-Perspective Pain and No Pain Conditions for Participants on the Bottom (Dark Purple) and Top (Light Purple) Median-Split Groups on the Callousness Factor*


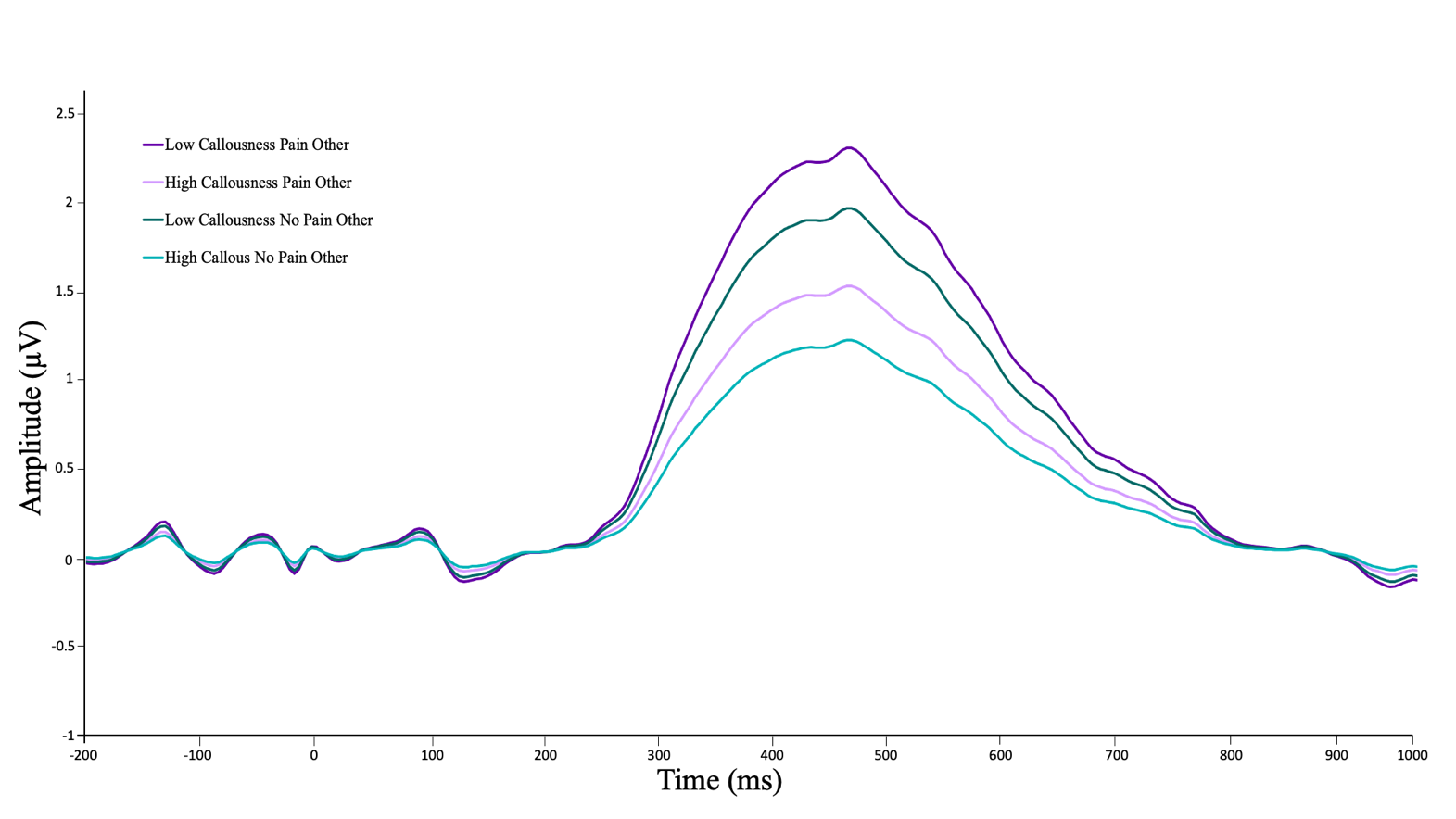


**2. Partial correlations between individual scale scores of callousness and LPP Pain Other controlling for Callousness factor scores**

To examine the extent to which scales contributing to the Callousness factor predicted unique variance in LPP reactivity to pain in others beyond the general factor explaining their covariance, a set of exploratory partial correlations were conducted. Residual LPP scores to pain in others were computed to account for its overlap with the other LPP conditions. Then, partial correlational analyses between each callousness scale scores (TriPM Meanness, LSRP Primary, ICU) and this residualized LPP variable were conducted controlling for Callousness factor scores. After controlling for Callousness factor scores, correlations between individual scale scores and LPP Pain Other amplitudes were: *r* = .04 for TriPM Meanness, *r* = -.08 for LSRP Primary, and *r* = .03 for ICU; all *p*s > .410. These results indicate that the observed relationship between Callousness factor scores and LPP Pain Other amplitudes was accounted for the shared variance between the three callousness scales.

**Supplemental References**

Dien, J. (2010). The ERP PCA Toolkit: An open source program for advanced statistical analysis of event-related potential data. *Journal of Neuroscience Methods*, *187*(1), 138-145. <https://doi.org/10.1016/j.jneumeth.2009.12.009>

Dien, J. (2012). Applying principal components analysis to event-related potentials: a tutorial. *Developmental Neuropsychology*, *37*(6), 497-517. <https://doi.org/10.1080/87565641.2012.697503>

Dien, J., Beal, D. J., & Berg, P. (2005). Optimizing principal components analysis of event-related potentials: matrix type, factor loading weighting, extraction, and rotations. *Clinical Neurophysiology*, *116*(8), 1808-1825. <https://doi.org/10.1016/j.clinph.2004.11.025>

Dien, J., & Frischkoff, G. A. (2005). Principal components analysis of ERP data. In T.C. Handy (Ed.), *Event-Related Potentials: A Methods Handbook* (pp. 189-207). Cambridge, MA: MIT Press.

Foti, D., Hajcak, G., & Dien, J. (2009). Differentiating neural responses to emotional pictures: Evidence from temporal‐spatial PCA. *Psychophysiology*, *46*(3), 521-530. <https://doi.org/10.1111/j.1469-8986.2009.00796.x>
